# The effects of HIV Tat DNA on regulating the immune response of HIV DNA vaccine in mice

**DOI:** 10.1186/1743-422X-10-297

**Published:** 2013-09-30

**Authors:** Ye Liu, Fusheng Li, Zhi Qi, Yanling Hao, Kunxue Hong, Yong Liu, Yulong Cong, Yiming Shao

**Affiliations:** 1 Department of Clinical Laboratory, Chinese P. L. A. General Hospital, No. 28 Fuxing Road, Beijing 100853, China; 2 State Key Laboratory for Infectious Disease Prevention and Control, National Center for AIDS/STD Control and Prevention, Chinese Center for Disease Control and Prevention, Collaborative Innovation Center for Diagnosis and Treatment of Infectious Diseases, 155 Changbai Road Changping District, Beijing 102206, China; 3 Statistical Center for HIV/AIDS Research and Prevention, Fred Hutchinson Cancer Research Center, 1100 Fairview Avenue N, Seattle, WA 98109, USA

**Keywords:** HIV DNA vaccine, Tat expression plasmid, T cell response, IgG subclass, Th polarization

## Abstract

**Background:**

HIV trans-activator protein (Tat) is the crucial factor to control HIV transcription, and is usually considered as an important immunogen for the design of HIV vaccine. Recent studies reported some special bio-activities of Tat protein on immunoregulation. However, to date, few studies have focused on exploring the effects of Tat expression plasmid (pTat) on regulating the immune responses induced by HIV DNA vaccines. In this study, our main objective is to investigate the immunoregulation mediated by pTat in mice.

**Methods:**

Four gene-coding plasmids (pTat, pGag, pEnv and pPol) were constructed, and the gene expression was detected by western blot method. The effects of pTat on regulating the immune responses to antigens Gag, Env, Pol were assessed by enzyme-linked immunospot and enzyme-linked immunosorbent assay. The data was analysed by one-way analysis of variance.

**Results:**

After two immunizations, mice vaccinated with antigen expressing plasmid (pGag, pEnv or pPol) plus pTat exhibited significantly stronger IFN-gamma response than that vaccinated with the corresponding antigen alone. Moreover, mice receiving two injections of antigen plus pTat exhibited the same strong IFN-gamma response as those receiving three injections of antigen alone did. Furthermore, addition of pTat not only induced a more balanced Th1 and Th2 response, but also broadened IgG subclass responses to antigens Gag and Pol.

**Conclusion:**

pTat exhibited the appreciable effects on modulating immune responses to HIV antigens Gag, Env and Pol, providing us interesting clues on how to optimize HIV DNA vaccine.

## Background

For a long time, traditional vaccines played the vital roles in controlling and eradicating infectious diseases that are life threatening, such as smallpox, cholera and polio [1]. In the early 1990s, Wolff and his colleagues directly injected mice with naked DNA plasmid, and found in surprise that antigen-specific T cell response could be induced by this simple way [2]. Further, they reported that the gene expression lasted for one year after intramuscular injection of the DNA plasmid [3]. From that time on, researchers redefined the nature of vaccine and realized that the genetic material coding antigen sequence could be used as the effective vaccine component.

DNA vaccines against human immunodeficiency virus (HIV) have been developed for nearly 20 years [4]. The characteristics of HIV DNA vaccines were also evaluated in many studies, indicating the good properties on safety, stability and easy-production [4-6]. Currently, one of the major challenges for HIV DNA vaccine development is its relatively modest immunogenicity in some animal models and in clinic trials [7]. To solve this problem, researchers tried a variety of methods, including developing cytokine genetic adjuvant such as IL-2, GM-CSF and IL-12, improving DNA delivery by gene gun or electrostimulation, modifying the properties of antigen presenting cells, and optimizing the antigen coding sequence to achieve higher gene expression level [8-14].

HIV Tat is the important transcription activation protein. It contains several distinct regions on the basis of its amino acid composition: N-terminal activation region, cysteine-rich role domain, core region, basic region and glutamine-rich region. Tat protein is encoded by two exons. The first exon is conserved in all viral isolates and encodes a 72-amino acid peptide which mediates the virus trans-activation [15]. The C-terminal amino acids which are encoded by the second Tat exon contain the arginine-glycine-aspartic acid (RGD) motif and mediate cell adhesion and binding of extracellular Tat [16].

In most cases, HIV-1 Tat is used as an immunogen [17,18]. However, the published data had also exhibited some other biological functions of Tat protein, such as inducing maturation of monocyte-derived dendritic cells (MDDCs), triggering a Th1-type dominant adaptive immune response, changing the subunit composition of the proteasome, modulating the humoral responses against unrelated antigens [19-22], and so on. Therefore, it may be hypothesized that Tat expression plasmid as a regulator modulates the immune responses against other crucial HIV antigens to optimize the efficacy of HIV vaccines.

In our current study, we constructed the Tat-expression plasmid (pTat) and added it directly into other HIV DNA vaccine component. By investigating the immune responses to HIV-1 antigens (Gag, Env and Pol), we found that pTat greatly improved T cell responses to these HIV antigens in mice, and such enhancement of antigen-specific T cell response by pTat was not caused by simply increasing the expression of target antigen . We also demonstrated that pTat could regulate Th skewed responses and broaden IgG subclass responses to antigens Gag and Pol. Collectively, our results suggested that pTat could effectively shape the immune responses triggered by HIV DNA vaccines.

## Results

### Effects of pTat on cellular immune responses to HIV antigens Gag, Env and Pol

**Figure 1:**
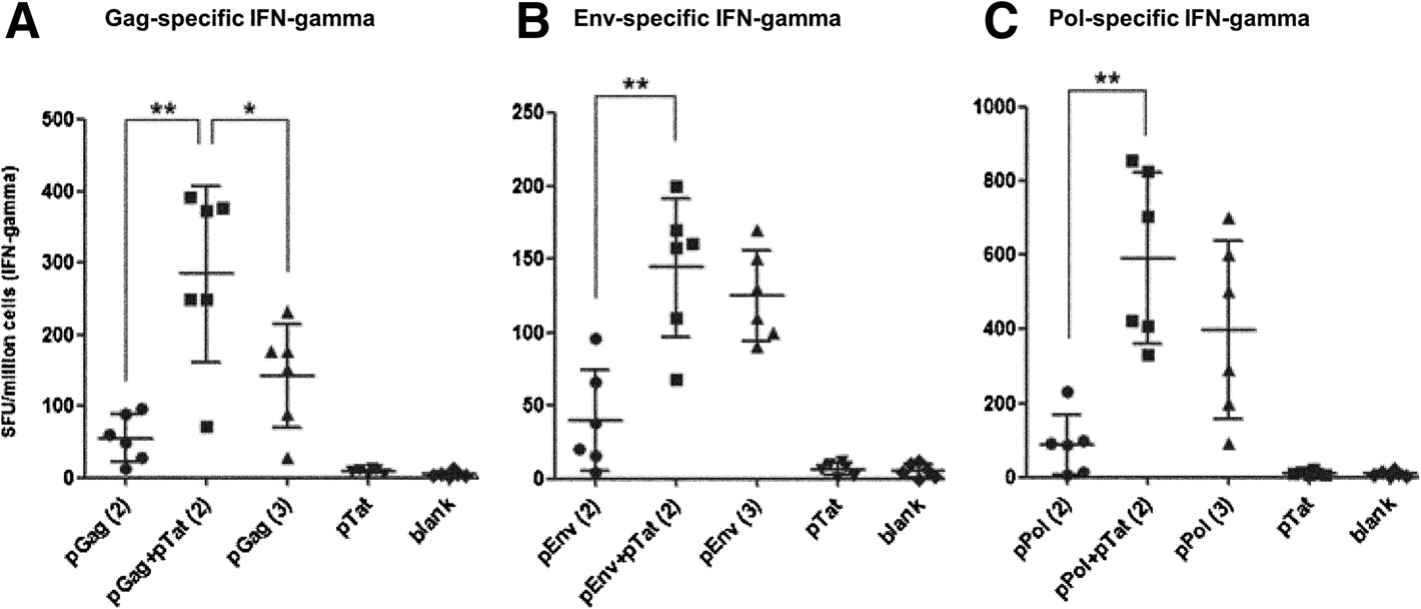
**Antigens**-**specific T cell responses.** In test groups, female BalB/C mice were intradermally vaccinated with pGag, pEnv or pPol, in the absence or presence of pTat. Blank plasmid group was used as negative control. pTat alone group was designed to test whether there are cross-reactive epitopes between Tat and HIV antigens Gag, Env and Pol (splenocytes from mice vaccinated with pTat alone were stimulated by Gag, Env and Pol peptides, respectively). Fresh splenocytes were stimulated by the corresponding positive peptides, and antigen-specifc IFN-gamma response was detected by ELISPOT assay. These experiments were repeated once. The data was shown as means ± SD. Part **A** showed the Gag-specific IFN-gamma response (splenocytes were stimulated by Gag peptide). “pGag(2)”: two immunizations with Gag expression plasmid alone (50 μg pGag and 50 μg empty vector each time); “pGag + pTat(2)”: two immunizations with the mixture of Gag expression plasmid and Tat expression plasmid (50 μg pGag and 50 μg pTat each time); “pGag(3)”: three immunizations with Gag expression plasmid alone (50 μg pGag and 50 μg empty vector each time); “pTat”: two immunizations with pTat (50 μg pTat and 50 μg empty vector each time); “blank”: two immunizations with empty vector (100 μg each time). Part **B** (Env-specific IFN-gamma response) and Part **C** (Pol-specific IFN-gamma response) can be done in the same manner. Symbol** means P < 0.01; Symbol* means P < 0.05.

In this study, we first investigated whether pTat could effectively enhance T cell responses to three HIV antigens (Gag, Env and Pol) in mice. The production of IFN-gamma by splenocytes from immunized mice was detected by enzyme-linked immunosorbent spot (ELISPOT) analysis. As shown in Figure 1A , after two intradermal vaccinations, pGag plus pTat (mixture of 50 μg pGag and 50 μg pTat) induced nearly six times stronger Gag-specific IFN-gamma response than that pGag alone did (mixture of 50 μg pGag and 50 μg blank plasmid). Using the same immunization protocol, significantly enhanced IFN-gamma responses to Env and Pol were also detected in mice vaccinated with pEnv + pTat and pPol + pTat respectively, compared with the mice vaccinated with pEnv and pPol alone (showed in Figure 1B and Figure 1C).

Subsequently, enhanced cellular immune responses to three HIV antigens by pTat were confirmed again in our “reducing immunization times” experiment. In the case of Env and Pol, mice receiving two vaccinations with pEnv plus pTat or pPol plus pTat produced the same high amount of IFN-gamma as those receiving three injections of pEnv or pPol alone, respectively (Figure 1B and Figure 1C). More encouragingly, two vaccinations with pGag plus pTat induced significantly stronger IFN-gamma response than three vaccinations with pGag alone did (p < 0.05, Figure 1A). These results suggested that addition of pTat could induce satisfied antigen-specific T cell response in the case of reducing immunization times. In other word, addition of pTat could shorten the immunization cycle effectively.

We also detected the production of IL-4 that was considered as one Th2-type cytokine for promoting B-cell maturation in all above-mentioned mice groups [23-25]. In contrast to the IFN-gamma responses, the ELISPOT results showed that neither pGag, pEnv and pPol stimulated the antigen-specific IL-4 production, nor the addition of pTat enhanced the secretion of IL-4 to a detectable level, (negative, data not shown).

### PTat failed to enhance HIV antigen expression *in vitro*

**Figure 2:**
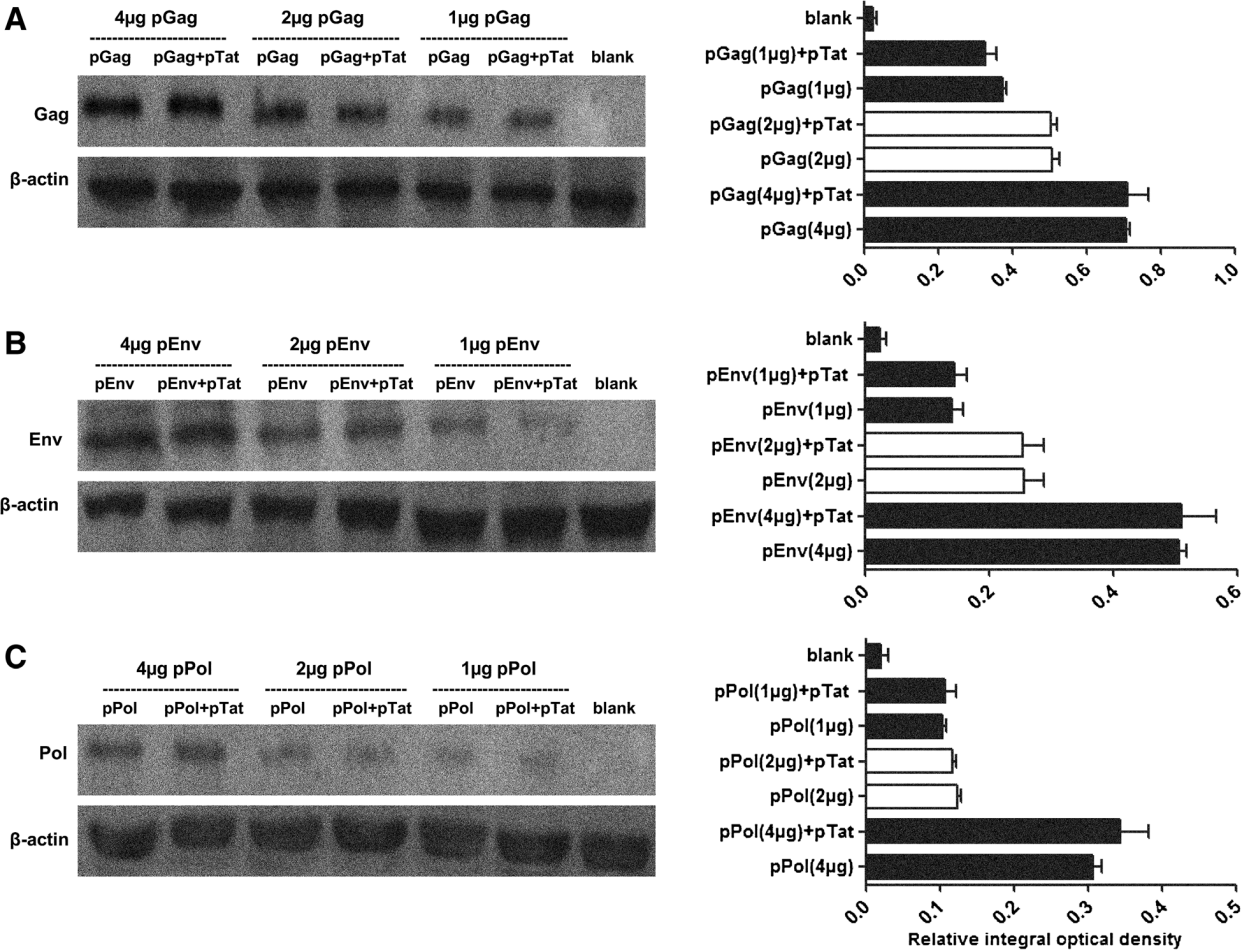
***In vitro *expressions of antigen Gag, Env and Pol.** 293T cells were prepared at a density of 1×10^6^ cells per well and 90% cell viability the night before transfection. Each plasmid (pGag, pEnv or pPol) was delivered to cells at three dose level (1 μg, 2 μg and 4 μg) respectively. Forty-eight hours after transfection, the expressions of antigens were detected by WB method, and the relative integral optical densities of antigens were measured (the average of two experiments with SD was indicated). Empty vector was used as blank control and β-actin was selected as internal control. Part **A**: the expressions of Gag were showed (left side): Gag-expression plasmids were delivered to 293T cells at three dose levels (1 μg, 2 μg and 4 μg) respectively, in the presence of pTat or in the absence of pTat. The expressions of Gag were quantified by detecting the relative integral optical densities of Gag antigen (right side): “pGag (1 μg)+pTat”, “pGag (2 μg)+pTat” and “pGag (4 μg)+pTat” means that pGag were delivered to cells at the dose of 1 μg, 2 μg and 4 μg respectively, in the presence of pTat. “pGag (1 μg)”, “pGag (2 μg)” and “pGag (4 μg)” means that pGag alone were delivered to cells at the dose of 1 μg, 2 μg and 4 μg, respectively. “Blank” means empty vector was delivered to cells. Part **B** (the expressions of Env) and **C** (the expressions of Pol) can be done in the same manner.

To understand the reasons leading to the increased antigen-specific IFN-gamma response mediated by pTat, we first investigated the effects of pTat on enhancing the expressions of HIV antigens Gag, Env and Pol. Serially diluted antigen expression plasmids, pGag, pEnv or pPol (1 μg, 2 μg and 4 μg for each plasmid), were transfected into 293 T cells in the absence or presence of pTat. Different amount of plasmid DNA was used in our transfection experiment to avoid the potential saturation effect. By comparing the values of relative integral optical density of target proteins, we found that, at the same dose of antigen expression plasmid, the expression of target antigen (Gag, Env or Pol) in 293 T cells which were transfected with antigen coding plasmid plus pTat was similar with that in cells receiving the corresponding plasmid alone. The results suggested that the expression of none of three HIV antigens (Gag, Env and Pol) was affected by the addition of pTat (Figure 2).

**Figure 3:**
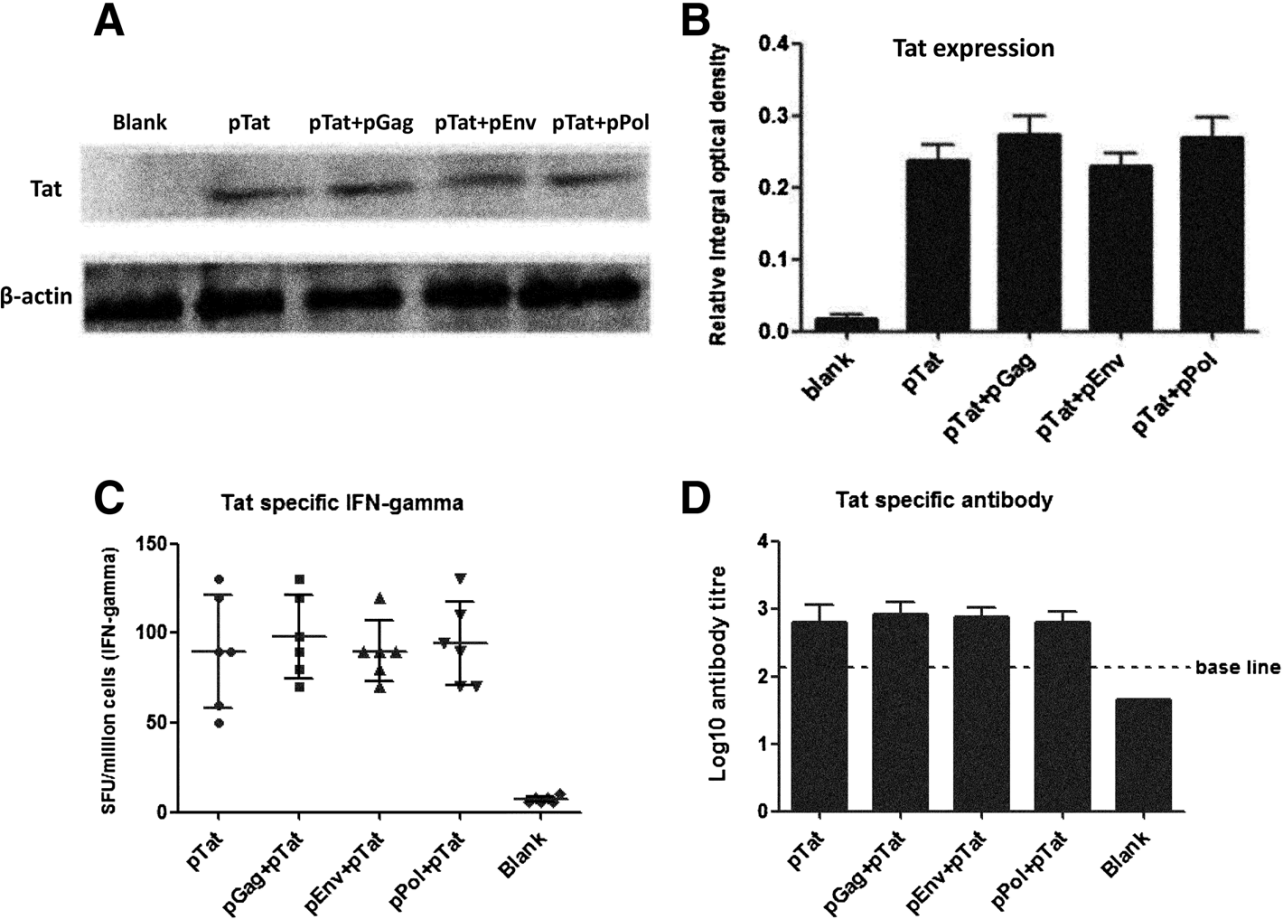
***In vitro *expression and immune response of Tat.** Part **A** and **B** showed the Tat expression in 293 T cells. Briefly, 293 T cells (1 × 10^6^ cells per well, and 90% cell viability at least) were transfected with pTat alone, pGag + pTat, pEnv + pTat, pPol + pTat and empty vectors, respectively. Forty-eight hours after transfection, Tat expressions in above-mentioned cell groups were detected by WB method (Figure 3A), and the relative integral optical density of Tat protein in each group was measured (Figure 3B). Empty vector transfection was used as blank control and β-actin was selected as internal control. In part **A** and **B**: pTat alone, pGag + pTat, pEnv + pTat, pPol + pTat means that 293 T cells received 4 μg pTat plus 4 μg empty vector, 4 μg pTat plus 4 μg pGag, 4 μg pTat plus 4 μg pEnv, 4 μg pTat plus 4 μg pPol, respectively. Blank: cells were transfected with 8 μg empty vector. Part **C** and **D** showed the Tat-specific immune responses. Female BalB/C mice were immunized twice. Two weeks after the final vaccination, mice were sacrificed and the spleens and blood were harvested. Tat-specific T cell and antibody responses were detected with IFN-gamma ELISPOT and IgG ELISA assay, respectively. In part **C** and **D**, pTat alone: mice received 50 μg pTat and 50 μg empty vector each time; pGag + pTat/ pEnv + pTat/ pPol + pTat: mice received 50 μg pGag/ pEnv/ pPol and 50 μg pTat each time; Blank: mice received 100 μg empty vector each time.

To explore whether no-expression of Tat protein (encoded by pTat) led to no-regulation of pTat on the expressions of three antigens (Gag, Env and Pol), we also detected Tat expression in 293 T cells which were transfected with pTat alone (4 μg pTat and 4 μg empty vector), pTat + pGag (4 μg pTat and 4 μg pGag), pTat + pEnv (4 μg pTat and 4 μg pEnv) and pTat + pPol (4 μg pTat and 4 μg pPol), respectively. The results indicated that the same strong Tat expression levels (detectable) were detected in these cell-transfection groups (as shown in Figure 3A and Figure 3B).

Together, our results demonstrated that pTat had no effects on increasing the *in vitro* expression of antigens Gag, Env and Pol, suggesting that enhanced IFN-gamma responses against target antigens were not due to that these antigens achieved the higher protein expression.

### No cross-reactive T cell epitopes between Tat and Gag, Env, Pol

In order to exclude the possibility that there are cross-reactive epitopes within Tat which were shared with Gag, Env or Pol, we prepared splenocytes from mice receiving two intradermal vaccinations with pTat alone (50 μg pTat each time), and stimulated them with Gag, Env or Pol peptides, respectively. The results showed that negative Tat-specific IFN-gamma responses were detected from splenocytes challenged with Gag, Env or Pol peptides (Figure 1A, Figure 1B and Figure 1C), suggesting that the cross-reaction between Tat and target antigens (Gag, Env or Pol) was not the reason for enhanced IFN-gamma response to HIV antigens by pTat.

### Effects of pTat on the total IgG responses to HIV antigens Gag, Env and Pol

**Figure 4:**
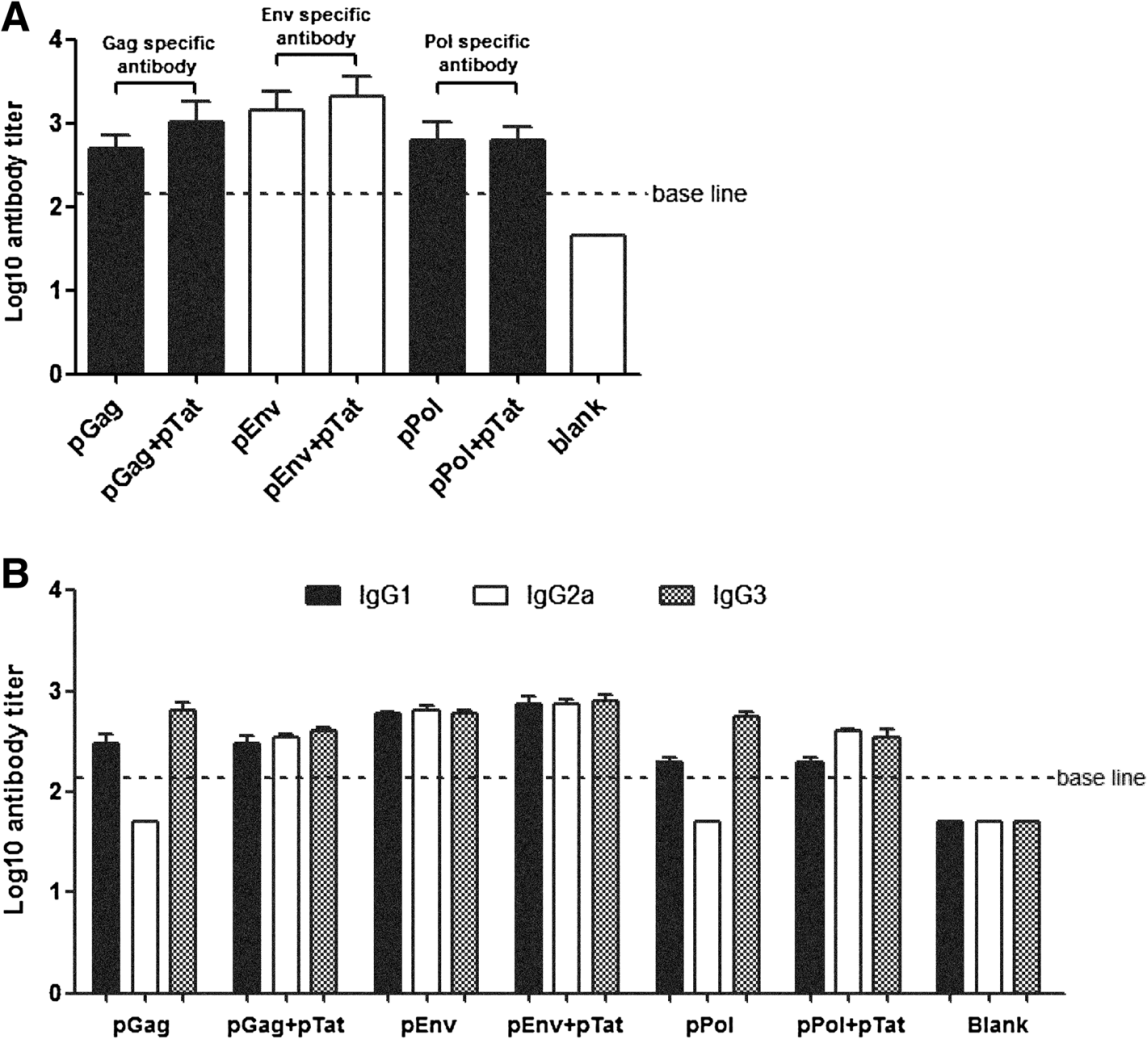
**Antigens-specific antibody responses.** Serum samples from mice vaccinated twice were harvested two weeks after the second immunization (The same mice as described in Figure 1). The titers of total IgG and three IgG subclasses (IgG1, IgG2a, IgG3) in each mouse serum were evaluated by standard ELISA assay (repeat once, 1:50 start dilution). The results are mean value (Log10 value) of each group of mice (n = 6) ± SD. Part **A** and Part **B** showed the total IgG and IgG subclasses responses to Gag, Env and Pol, respectively. pGag/ pEnv/ pPol: two immunizations, 50 μg pGag/ pEnv/ pPol and 50 μg empty vector each time; pGag + pTat/ pEnv + pTat/ pPol + pTat: two immunizations, 50 μg pGag/ pEnv/ pPol and 50 μg pTat each time.

We then evaluated the effects of pTat on the total IgG responses to HIV antigens Gag, Env and Pol by measuring IgG titers in serum samples from vaccinated mice (the same mice as those described in Figure 1 by enzyme-linked immunosorbent (ELISA) assay. As showed in Figure 4A, the results showed that pPol alone induced the same strong IgG response as vaccination with pPol plus pTat did in mice. Similarly, compared with pGag-vaccination alone, pGag plus pTat only induced the limited enhancement of Gag-specific IgG titer, which was not statistically significant (*p* > 0.05). The same result was also exhibited in the case of pEnv. Collectively, these results suggested that pTat had limited effect on boosting the total IgG responses to HIV antigens Gag, Env and Pol.

### Effects of pTat on IgG subclass production

We further investigated the effects of pTat on IgG subclass production by measuring the titers of three IgG subclasses (IgG1, IgG2a and IgG3) in mice serum samples. As shown in Figure 4B, after two intradermal immunizations, both pEnv alone and pEnv plus pTat induced positive IgG1, IgG2a and IgG3 responses to Env. In the case of Gag and Pol, positive IgG1 and IgG3 responses to Gag and Pol were detected in all mice groups receiving pGag and pPol respectively, regardless of the presence or absence of pTat. In contrast, positive IgG2a responses to Gag and Pol were detected only in mice immunized with pGag plus pTat and pPol plus pTat, but not in mice immunized with pGag and pPol alone. These results suggested that pTat broadened the IgG subtype responses to Gag and Pol.

### Effects of pTat on Th polarization

We then compared the IgG1 (Th2 polarization) and IgG2a (Th1 polarization) subclass responses generated by three antigen expressing plasmids (pGag, pEnv and pPol) immunizations with or without pTat, respectively. By calculating Th1:Th2 index according to the formula of “IgG2a/IgG1”, we found that the Th1:Th2 index in mice vaccinated with pGag, pEnv and pPol alone were 0.17, 1.08 and 0.25, respectively (Table 1), indicating that pGag and pPol alone induced Th2-polarized antibody response in mice while pEnv alone induced a balanced Th1/Th2 response. Nevertheless, addition of pTat turned the Th2 dominant antibody responses to Gag and Pol into the more balanced Th1/Th2 responses (Th1:Th2 index for Gag and Pol was 1.17 and 2, respectively). Therefore, the results suggested that pTat had effects on shaping Th polarization induced by HIV DNA vaccines pGag and pPol.

**Table 1:** Th polarization

	**pGag (titer)**	**pGag + pTat (titer)**	**Blank (titer)**
**IgG1 (Th2)**	300	300	50 (negative)
**IgG2a (Th1)**	50 (negative)	350	50 (negative)
**Th1:Th2 index**	0.17	1.17	--
	**pEnv (titer)**	**pEnv + pTat (titer)**	**Blank (titer)**
**IgG1 (Th2)**	600	750	50 (negative)
**IgG2a (Th1)**	650	750	50 (negative)
**Th1:Th2 index**	1.08	1	--
	**pPol (titer)**	**pPol + pTat (titer)**	**Blank (titer)**
**IgG1 (Th2)**	200	200	50 (negative)
**IgG2a (Th1)**	50 (negative)	400	50 (negative)
**Th1:Th2 index**	0.25	2	--

The mean value of each group of mice (the same data as showed in Figure 4B) was showed in this table, and used to calculate the index of IgG2a/IgG1 [26]. Th-polarization response in mice was determined according to the value of IgG2a/IgG1 [27,28]. The value of Th1:Th2 are accurate to two decimal places. All experiments were repeated once, and the results were consistent.

### Tat-specific immune responses in mice

Tat-specific immune responses were also detected in our current study. After two immunizations, mice vaccinated respectively with pTat alone, pTat plus pGag, pTat plus pEnv, pTat plus pPol and empty vectors were sacrificed, and the splenocytes and serums were harvested for ELISPOT and ELISA assay. As shown in Figure 3C, all four mice groups (pTat alone, pTat plus pGag, pTat plus pEnv, pTat plus pPol) exhibited the positive Tat-specific T cell response, but no significant differences among these test groups (P > 0.05). In the case of antibody response against Tat, similarly strong antibody responses (positive) were also detected in all four pTat-immunization mice groups (Figure 3D). The results indicated that pTat could induce the positive T cell and antibody responses to Tat itself. Moreover, it also might suggest that pGag, pEnv and pPol had no influences to the immune response against HIV Tat.

## Discussion

As an important regulator in the early stage of HIV infections, Tat protein mainly exerts its biological function on controlling HIV transcription when the proviral genome is transported to the nucleus and integrated into the host cell genome [29]. However, in the recent ten years, accumulating studies have showed some other natural bioactivities of Tat protein on shaping immune characters of other antigens, providing us clues for designing new vaccines against AIDS.

In fact, Tat protein as a vaccine component had been used in clinic trial, and exhibited some special effects on optimizing vaccine efficacy. Ensoli *et al*. in their Phase II clinical investigation demonstrated that therapeutic immunization with Tat induced a safe and durable immune response, modified the pattern of CD4^+^ and CD8^+^ cellular activation, increased the T cell response against Env, as well as effectively intensified HAART efficacy and restored immune homeostasis, providing the encouragement for combining Tat immunization with conventional virus-targeting drugs for an improved treatment of HIV disease [30]. In animal models, Tat protein as immunogen or regulator had been assessed more completely. For example, Florese J *et al*. reported for the first time that Tat protein served as a target for ADCC and Tat specific antibody mediated ADCC killing in macaques [31]. And Tat protein was also found to possess auto-adjuvanticity to raise an adjuvant-free humoral immune response against Tat itself controlled by its core region in mice [32]. Other researchers explored the effects of Tat on favoring protective immunity against Leishmania major in mouse model [33]. Moreover, the HIV vaccines based on Tat were also demonstrated to be immunogenic and be able to protect macaques from mucosal or intravenously simian/human immunodeficiency virus (SHIV) challenge [34,35].

To date, most investigations against the regulation of HIV Tat on immune responses induced by vaccines were based on Tat protein, and few studies reported the regulation activities of Tat DNA, especially Tat expression plasmid. Actually, Tat expression plasmid (pTat) itself possesses some special advantages, compared with Tat protein or virus vector encoding Tat: (1) pTat could be administrated repeatedly *in vivo* without having to consider to elicit the anti-vector immune responses in the process of vaccination; (2) commercialized production of pTat is relatively inexpensive; (3) pTat itself is stable and convenient for transportation. Therefore, Tat-expression plasmid as a vaccine component should be an appropriate candidate. In our current study, we demonstrated that pTat which encodes the full length Tat gene (contains two exons, 101 amino acids) could enhance IFN-gamma responses against three vital antigens (Gag, Env and Pol) used in HIV vaccines. Moreover, the enhancement of Gag-specific T cell response caused by addition of pTat was proved to be better than that caused by linking ubiquitin to Gag (data not shown), which was considered as an effective strategy for improving antigen-specific cellular immune response in many foregoing studies [36,37], suggesting the excellent capability of pTat on enhancing IFN-gamma production. Similar enhancement of Gag-specific T cell response by Tat was also reported in Zhao *et al*. paper which showed that co-delivering of Tat and Gag with the Ad5hr vector enhanced Gag-specific IFN-gamma response [38]. Moreover, Gavioli *et al*. found that Tat protein as a novel Th1-type adjuvant had the property of broadening and enhancing T cell responses to HIV structural antigens (Env and Gag) and unrelated antigen (ovalbumin) in mice [39], via modifying the composition of the proteasome and its enzymatic activities [21,40]. Such enhancement of IFN-gamma response to crucial antigens (such as Gag, Env and Pol) is considered to be meaningful for optimizing the efficacy of HIV vaccine. Our previous research showed that HIV-specific T cell IFN-gamma response is associated with seronegative status in highly exposed subjects in China, suggesting that a strong IFN-gamma immunity against HIV may be helpful to prevent the infection or control the progression of HIV [41]. To understand the reasons of the enhancement of T cell responses to other HIV antigens caused by pTat, we first investigated whether pTat would up-regulate these antigen expressions. The WB results showed that pTat failed to promote the expressions of other HIV antigens *in vitro*. Moreover, we also excluded the possibility that cross-reactive epitopes within Tat which were shared with Gag, Env and Pol resulted in the enhancement of T cell responses against Gag, Env and Pol. Of note, some previous studies provided us more clues to explore the mechanism how Tat enhanced T cell responses against other antigens. Barbara Ensoli group demonstrated that the enhanced capability of DCs in capturing antigens caused by Tat was the major factor leading to the enhanced antigen-specific T cell responses. Specifically, Tat was able to stimulate the maturation of CD1a-expressing MDDCs, improve the capability DCs in capturing antigens, up-regulate MHC and costimulatory molecules, as well as induce high cytokine production [20]. In addition, the study from Guoqing Zang group showed that the protein transduction domains (PTD) of HIV Tat protein could interact with exogenous antigens and help them enter cells, resulting in increased cytokine production [42]. In contrast, Shalini Gupta *et al*. found that Tat suppressed the cellular immune response to HIV Env/gp120 in mice when gp120 and Tat were delivered by the bicistronic expression vector, since Tat induced strong secretion of IL-10 which has appreciable T cell inhibitory activity [43]. Similar inhibition was also found in the study by Mooij *et al*., showing that immunization with combined antigens (Tat-Gag-Env) reduced the magnitude of the response to Tat compared to the single-antigen immunization [44].

Regarding the effects of pTat on modulating humoral immune responses, our results showed that pTat had no effects on increasing the total IgG titer against Gag, Env and Pol in mice. It was inconsistent with the result from another group: they found that the auto-adjuvanticity of Tat protein could be transferred to unrelated antigens and enhanced these antigens specific humoral immune responses [22]. Meanwhile, the humoral response characterized by the broad anti-HIV IgG subclasses was demonstrated to be associated with the long-term no progress (LTNP) status in some previous clinical reports [30,45,46]. Therefore, we further investigated the IgG subclass responses in mouse serum samples. The results showed that, compared with the mice vaccinated with pGag or pPol alone, the broader IgG subclass responses to Gag and Pol were induced upon the addition of pTat. Moreover, our result also indicated that pTat could turn the Th2-polarization responses induced by pGag and pPol alone to the more balanced Th1/Th2 immune responses in mice. Similar results were reported in the study by Kulkarni *et al*., which showed Tat modulated Th1 differentiation as well as the class switch recombination to IgG2a of B cells via up-regulating the transcription factor T-bet [47].

Besides, biosafety is another major concern needed to be considered during the DNA vaccine design. Our plasmid backbone was optimized to minimize the potential adverse effects by deleting unnecessary prokaryotic elements or replacing those functional regions derived from prokaryotic genes [48]. Moreover, the antigens used in our current study have also been modified by deleting or mutating the toxic sites within the antigen-coding genes. More importantly, the DNA vaccines used in our current research have already entered Phase I clinic trials after receiving satisfied safety assessment from the good laboratory practice toxicology study in animal models (data not shown).

Finally, it should be noted that the enhancement of T cell response by Tat in this study was tested in BalB/C mice. Some published data showed that the pattern of Th1/Th2 response induced by Tat protein was different between mice and cynomolgus macaques [49]. Therefore, whether a similar effect can be observed in nonhuman primates and humans needs to be determined in further experiments. Considering the significant enhancement of T cell responses and the broadened range of IgG subclass production achieved by pTat, our study provided a new strategy to optimize the efficacy of HIV DNA vaccine.

## Conclusion

In this study, HIV Tat expression plasmid significantly enhanced IFN-gamma responses to crucial antigens (Gag, Env, Pol) used in HIV vaccines in mice. Moreover, it also has been found to broaden IgG subclass responses to Gag and Pol, and induce a more balanced Th1 and Th2 response.

This study demonstrated the effects of Tat expression plasmid on modulating immune responses to other HIV antigens, and provided the evidence that Tat expression plasmid as a regulator could be used to enhance the efficacy of HIV DNA vaccine. The present study raises the possibility that Tat expression plasmid could be further developed into a novel vaccine component to optimize HIV vaccines in clinical trials.

## Methods

### Ethics statement

Animal experiments were approved by the Animal Ethics Committee of Chinese P. L. A. General Hospital, and were carried out in accordance with the guidelines of the Beijing Municipality on the Review of Welfare and Ethics of Laboratory Animals. Mice were anesthetized with Zoletil 50 (Virbac, SanteAnimale) in phosphate-buffered saline (PBS) by subcutaneous administration immediately prior to vaccination or sacrifice.

### Consents

Written informed consent was obtained from the patient for the publication of this report and any accompanying images.

### Plasmids construction

**Figure 5:**
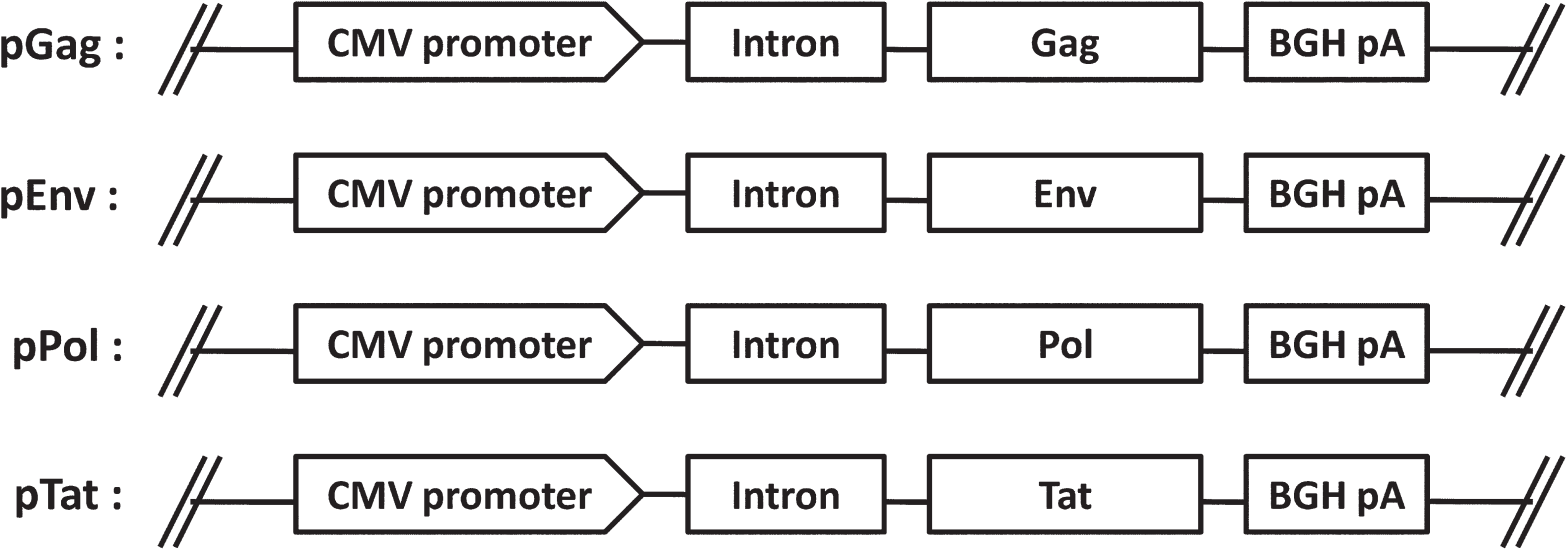
**Schematic maps of HIV genes expression plasmids.** pGag, pEnv, pPol and pTat means the plasmid encoding Gag, Env, Pol and Tat protein, respectively. CMV, human cytomegalovirus (hCMV) immediate/early (IE) promoter; Intron, hCMV IE intronA; BGH pA, bovine growth hormone polyadenylation signal.

Expression plasmid pDRVI1.0 is an optimized mammalian expression vector constructed by Prof Yiming Shao group. It contains intronA sequence of CMV promoter and BGH polyA signal [50,51]. Genes coding Gag, Env, Pol or Tat were obtained from HIV-1 CN54 (a CRF07_BC strain) and cloned into pDRVI1.0 separately by standard method. All four constructs (pGag, pEnv, pPol and pTat showed in Figure 5) were verified by restriction enzyme analysis and sequencing, and purified by Qiagen (Va-lencia CA) endotoxin-free columns.

### Western blot (WB) analysis

293 T Cells (ATCC, CRL-11268) cultured with DMEM containing 10% fetal bovine serum were seeded in six-well plate at a density of 1 × 10^6^ cells/well and at least 90% cell viability. The cells were transfected with one kind of antigen-expression plasmid (pGag, pEnv or pPol) in the absence or presence of pTat using lipofectamine-2000 (Invitrogen, Carlsbad, CA) at a ratio of 1.5 μl lipofectamine-2000 to 1 μg plasmid DNA. Each antigen-expression plasmid was delivered to 293 T cells at three dose levels (1 μg, 2 μg and 4 μg) respectively. Appropriate dose of blank plasmid was added into the transfection wells to ensure that the cells per well received the same amount of plasmid. The cells were harvested 48 hours after transfection and lysed with lysis buffer (20 mM Tris–HCl, pH7.5, 150 mM NaCl, 1 mM EDTA-Na_2_, 1 mM EGTA, 1% Triton, 2.5 mM sodium pyrophosphate, 1 mM β-glycerophosphate, 1 mM Na_3_VO_4_, 1 μg/ml leupeptin). PIC and 1 mM PMSF were added into lysis buffer to prevent protein degradation. Cell lysates were centrifuged at 14,000 × g for 10 minutes, and the proteins in supernatants were boiled for 5 minutes, separated by 12% SDS-PAGE and transferred onto PVDF membranes. The membranes were blocked with 5% skimmed milk in Tris-buffered saline with 0.05% Tween20 (TBST) for one hour at room temperature and incubated with primary antibodies (Positive IgG response serums from mice vaccinated with pTat, pGag, pEnv or pPol respectively; Rabbit anti-β-actin polyclone antibody purchased from Santa Cruz Biotechnology, Santa Cruz, CA) at 1:500 dilution at 4°C overnight. The membranes were then washed with TBST, incubated with secondary goat-anti-rabbit antibody or goat-anti-mouse antibody coupled to horseradish peroxidase (HRP) (Santa Cruz Biotechnology, Santa Cruz, CA) at 1:2000 dilution for one hour at room temperature, and developed with chemiluminescent detection reagent. Finally, WB membranes were scanned and quantified using the Gel/Chem doc program Quantity one (Bio-Rad, Milan, Italy). Protein expression levels were obtained from images using the Quantity One software (v.4.5.1; Bio-Rad).

### Mice immunization

Female BalB/C mice of six to eight weeks old were obtained from the Laboratory Animal Center in the Academy of Military Medical Sciences in China. The mice were randomly divided into eleven groups with six mice per group: pGag alone (two injections), pGag alone (three injections), pGag plus pTat (two injections), pEnv alone (two injections), pEnv alone (three injections), pEnv plus pTat (two injections), pPol alone (two injections), pPol alone (three injections), pPol plus pTat (two injections), pTat alone (two injections) and blank (two injections). Purified plasmids were reconstituted in endo-free H_2_O for intradermal immunization. The dose of each plasmid DNA was 50 μg per mice each time. 50 μg empty vector was used in mice which were vaccinated with only one plasmid DNA to ensure all mice received a total of 100 μg plasmid. The spacing interval between two vaccinations was three weeks. Two weeks after the final vaccination, mice were sacrificed and the spleens and blood were harvested. Fresh splenocytes were prepared for ELISOPT assay and serum samples were prepared for ELISA assay.

### Enzyme-linked immunosorbent spot (ELISPOT) assay

ELISPOT assay was finished using commercial kits from BD Pharmingen (mouse IFN-gamma and IL-4 ELISPOT sets). Briefly, cytokine capture antibody against mouse IFN-gamma or IL-4 (1 μl/200 μl sterile PBS) was coated onto PVDF in 96-well plates by overnight incubation at 4°C. The plates were blocked with complete 1640 containing 10% FBS for two hours at room temperature. Fresh prepared mice splenocytes (5 × 10^5^ cells/well) were added into the plates immediately after the addition of 5 μg/ml specific H-2d-restricted CD8^+^ peptides (peptide sequences: Gag, AAMQILKDTINEEAA; Env pool, DTEVRNVWATHACVPADPNP, SELYK- YKVVEIKPLGVAPTT, QQSNLLRAIEAQQHLLQLTV; Pol pool, GTVLVGPTPV- NIIGR, VGPTPVNIIGRNLLT, HGVYYDPSKDLIAE, YYDPSKDLIAEIQKQ; Tat, IFYGRKERRQERSAH). The plates were incubated for 24 hours at 37°C with 5% CO2, and washed four times with PBST (0.05% Tween20). Then, the plates were incubated with 2 μg/ml biotinylated detection antibody against mouse IFN-gamma or IL-4 for two hours. ELISPOT development was performed by one hour incubation with avidin-HRP complex in PBST, followed by washing four times with PBS. Finally, the plates were incubated with peroxidase substrate AEC for 30 minutes. ELISPOT spots were measured with the automated ELISPOT Reader System (Bio-Red). For each mice group, the cut-off value to consider a positive response by ELISPOT was that: (1) the number of specific spots per well had to be at least two times the average value found in negative control wells; (2) the average value in negative control wells had to be not more than 20 spot forming units/SFU per million splenocytes; (3) the response had to be higher than 50 SFU per million splenocytes [52,53]. Splenocytes from mice vaccinated with empty vectors were used as negative/blank controls.

### Enzyme-linked immunosorbent (ELISA) assay

ELISA assay was performed to measure antibody titers in mouse serum samples. 96-wells flat bottom plates (Costar, Corning, NY) were coated with one of the three purified recombinant proteins (Gag/P24 protein, Env/gp120 protein, Pol/P51 protein; These proteins were home-made in E.coil expression system, and derived from a major epidemic strain in China, CN54, which is derived from the Chinese isolate 97CN001 which is a B/C recombinant strain. The purity of target proteins is >90%) at a concentration of 0.01 μg/ml in coating buffer (0.012 mol/L Na_2_CO_3_, 0.038 mol/L NaHCO_3_, pH9.6) at 4°C overnight. The plates were washed five times with PBST, and blocked with 3% BSA in PBST at 37°C for one hour. Mouse serum samples were diluted with blocking solution, and added into each well (100 μl/well). After incubation at 37°C for one hour, the plates were washed five times with PBST and then incubated with 1:5000 diluted HRP-labeled antibodies against mouse IgG, IgG1, IgG2a or IgG3 (Santa Cruz Biotechnology) at 37°C for one hour. The plates were then washed five times with PBST. After the final wash, 100 μl fresh-prepared TMB substrate solution (Sigma, St. Louis, MO) was added into each well, and the plates were incubated for 5 minutes. The reaction was stopped by addition of 25 μl 2 M H_2_SO_4_. The optical density (OD) was measured at 450 nm by Multiscan enzyme-linked immunosorbent assay plate reader (Thermo Life Sciences, Hampshire, United Kingdom). The cut-off value was determined: (1) a OD450nm value >0.1 at the dilution of 1:100 (if not, the sample was considered as negative) and (2) at least three folds that of the negative control were considered as positive [54,55]. Endpoint titers were expressed as Log10 concentrations. The serum samples from empty vector vaccination mice were considered as negative control.

### Th1:Th2 index calculation

Th1:Th2 index was calculated as previously described [27,28]. Specifically, antibody titers above 100 were considered positive whereas antibody titers under 100 were considered negative and excluded from the data analysis. In each group, the average value of antibody titer at the last positive dilution for each IgG subclass was used. Th1:Th2 index was calculated according to the formula of IgG2a/IgG1.

### Statistical analysis

Values were expressed as means ± standard deviations (SD). Analysis of differences in means between groups was conducted by one-way analysis of variance (ANOVA); *P* < 0.05 was considered significant.

## Competing interests

The authors declare that they have no competing interests.

## Authors’ contributions

Conceived and designed the experiments: YMS YLC YL. Performed the experiments: YL. Analyzed the data: YL ZQ. Wrote the paper: YL FSL YLH KXH YL. All authors approved the final manuscript.
